# Novel Quantitative Metrics for Foveal Hypoplasia Derived from Optical Coherence Tomography Angiography Using AngioTool

**DOI:** 10.3390/jcm15176724

**Published:** 2026-08-29

**Authors:** Charlotte Egbring, Sarah Kleemann, Nicole Eter, Jens Julian Storp

**Affiliations:** Department of Ophthalmology, University of Muenster Medical Center, 48149 Muenster, Germany; sarah.kleemann@med.uni-muenchen.de (S.K.); nicole.eter@ukmuenster.de (N.E.); jens.julian-storp@augen-am-meer.de (J.J.S.)

**Keywords:** FH, fovea plana, foveal pit, Leicester, angiogram, flow density, FD, OCT-A, SCP, DCP

## Abstract

**Background/Objectives**: This monocentric case–control study prospectively recruited patients with foveal hypoplasia (FH) and compared them with retrospectively selected age- and sex-matched healthy controls. It is the first study to employ AngioTool software to quantitatively assess retinal microvascular alterations in FH using Optical Coherence Tomography Angiography (OCT-A). **Methods**: In total, 19 patients with FH and 19 age- and sex-matched healthy controls were included at Münster University Hospital, Germany, and underwent OCT-A imaging of the macula. The 3 × 3 mm^2^ angiograms of the superficial (SCP) and deep capillary plexus (DCP) were examined using AngioTool software. Quantified parameters included flow density (FD), number of junctions, total and average vessel length, number of endpoints, and lacunarity. **Results**: A total of 62 eyes from 38 participants were included in the analysis. Compared with controls, eyes with FH demonstrated significantly lower FD, fewer junctions, shorter total vessel length, and a greater number of endpoints in both SCP and DCP. These differences showed small-to-large effect sizes, depending on the parameter and plexus. Average vessel length remained significantly decreased in the SCP but not the DCP. The difference in SCP lacunarity did not remain significant after adjustment for multiple comparisons. In the exploratory subgroup analysis, there were no significant differences between mild and severe FH in either the SCP or the DCP after correction for multiple testing. **Conclusions**: FH is associated with alterations of retinal microvasculature involving both the SCP and DCP. These findings are consistent with altered retinal vascular development in FH. OCT-A may provide useful quantitative information for the characterization of FH, although the clinical and prognostic relevance of these vascular parameters remains to be established.

## 1. Introduction

Foveal hypoplasia (FH) represents an uncommon condition that is characterized by the absence of the foveal pit or its incomplete development. Most affected individuals have subnormal visual acuity (VA) [1]. The most frequent genetic cause of FH is albinism [2], followed by gene variants that can be inherited autosomal dominantly [3], autosomal recessively [4,5] or in an x-linked inheritance [6,7]. Prematurity is regarded as the most relevant risk factor, largely due to the abnormal retinal vascular development and macular hypopigmentation that occur in these patients [8].

The ocular phenotype is typically characterized by subtle pigmentary alterations and the absence of a foveal light reflex on funduscopic examination. In fluorescein angiography (FA), FH is characterized by the persistence of retinal vessels within the foveal center and the absence of a foveal avascular zone (FAZ) [9]. Optical coherence tomography (OCT) has further demonstrated that the fovea centralis in FH remains anatomically underdeveloped.

OCT-angiography (OCT-A) has recently emerged as a non-invasive imaging technique that enables visualization and quantification of retinal microvasculature while simultaneously providing cross-sectional structural information [10]. This method has proven especially useful in delineating disorders with altered retinal capillary structures [11]. A detailed understanding of retinal microvascular alterations in FH is important for clarifying the underlying pathophysiology of the condition. Because of the rarity of FH and the relatively recent availability of OCT-A, most published studies have been limited to single case reports [12,13,14] or small case series involving two [15,16] or at most three patients [17,18,19], providing qualitative rather than quantitative information.

Previous quantitative studies have demonstrated FH patients to exert a global decrease in flow density (FD) in whole en face scans, whereas the foveal sector of the inner retinal plexus displayed increased FD values [20,21]. Further investigations of macular subregions, deeper vascular layers, and FAZ morphology revealed significantly increased FD values in the foveal areas of both the superficial and deep retinal capillary plexus (SCP and DCP), together with distinct alterations in FAZ size, perimeter, and acircularity index (ACI) when compared with controls, without clear correlation to disease severity [22]. Interestingly, the degree of DCP involvement was shown to correlate both with VA and with FH severity [23].

A common feature across all these studies is the evaluation of FD in different macular subregions and the assessment of FAZ morphology. However, a comprehensive investigation of the vascular plexuses themselves remains scarce in the literature. AngioTool enables the determination of a wide range of vascular parameters [24] and has already been applied to OCT-A images in other macular vascular diseases, including macular telangiectasia type 2 [25,26]. However, to our knowledge, its application to the quantitative assessment of retinal vascular architecture in FH has not been previously reported. A precise characterization of vascular changes in FH is crucial, as it may provide valuable insights into disease mechanisms and inform prognosis and therapeutic strategies.

The present study therefore aims to quantitatively assess microvascular parameters of the SCP and DCP in eyes with FH compared to healthy controls, using OCT-A in combination with advanced image analysis software. In addition, the influence of FH severity was evaluated. The main objective is to characterize layer-specific capillary adaptations in FH in order to firstly improve our understanding of the vascular changes occurring in FH and to secondly explore their clinical relevance.

## 2. Materials and Methods

This monocentric study was approved by the ethics committee of the Medical Association of Westfalen-Lippe and the University of Münster (reference: 2024-332-f-S). It adhered to the principles of the Declaration of Helsinki and included patients with FH who attended the Department of Ophthalmology at the University of Münster Medical Centre between 4 October 2024 and 31 July 2025.

In addition, a retrospectively selected group of healthy individuals was included to serve as a control cohort. Potential control subjects were identified from the institutional imaging database and screened for eligibility based on their available ophthalmological and medical records. Control subjects were required to have an unremarkable fundus examination and no evidence or history of ocular disease, no previous ocular surgery or retinal surgery, no relevant media opacities affecting retinal imaging, no diagnosis of diabetes mellitus, and no history of smoking. Eligible control subjects were individually matched to FH patients for sex and age, with a maximum age difference of three years, and were required to meet the same imaging quality criteria as the FH cohort. Written informed consent was obtained from all participants before enrollment.

All participants underwent a standardized ophthalmological examination, which comprised anterior segment evaluation, fundus examination, assessment of refractory status, and measurement of intraocular pressure. Subjects with high myopia, defined as a spherical equivalent of ≤−6.00 diopters, were excluded from participation. For the FH cohort, an additional prerequisite was the absence of retinal disorders other than FH. To minimize potential confounding factors, both FH patients and controls were required to have no relevant media opacities affecting retinal imaging, no history of retinal surgery, no diagnosis of diabetes mellitus, and no history of smoking.

For the diagnosis of FH, spectral-domain OCT (SD-OCT; Spectralis OCT, Heidelberg Engineering, Heidelberg, Germany) was employed to obtain cross-sectional retinal images. OCT examinations were performed using the Heidelberg Eye Explorer 2 (HEYEX 2) platform with SPECTRALIS Software Version 7.4 (Heidelberg Engineering, Heidelberg, Germany). FH was classified according to the Leicester Grading System for Foveal Hypoplasia [27]. According to this scheme, lower stages (1a–2) retain outer nuclear layer (ONL) widening and outer segment (OS) lengthening, with the foveal pit being shallow or absent, whereas higher stages (3–4) are characterized by the successive loss of these features in addition to the missing foveal pit. The classification has been validated across multiple studies [2] and was recently shown to provide prognostic value for visual development in affected children [28].

Throughout this manuscript, the term FH is used to describe the developmental disorder characterized by incomplete foveal maturation according to the Leicester grading system. Although the term “fovea plana” is sometimes used in the literature to describe the absence of a foveal pit, it does not necessarily encompass the full spectrum of structural abnormalities observed in FH.

After SD-OCT acquisition, OCT-A examinations were performed in the same darkened, windowless room with the RTVue XR Avanti system (Angiovue/RTVue-XR Avanti OCT, Optovue Inc., Fremont, CA, USA) without pharmacological pupil dilation. OCT-A imaging was performed using the AngioVue software algorithm, Version 2018.1.0.43 (Optovue Inc., Fremont, CA, USA). Macular angiography was performed using 3 × 3 mm^2^ scans, providing en face images of both the SCP and DCP. To minimize the potential influence of fluctuations in blood pressure or heart rate, patients were instructed to rest for five minutes before imaging [29].

Accurate localization of the foveal center in FH eyes posed a challenge for automated imaging. Although the OCT-A device employed an integrated tracking and follow-up system to delineate the FAZ and align the measurement grid with the central macula, the software frequently failed in advanced FH stages, resulting in misaligned grids. To overcome this limitation, the grid was manually adjusted using real-time infrared reference images. The fovea was subsequently identified by analyzing the vascular architecture on angiograms, specifically the convergence of major retinal vessels toward the center. Correct grid placement was confirmed in this manner. The evaluation of OCT-A scans was conducted independently by two ophthalmologists with extensive experience in this technology. In cases of disagreement regarding foveal localization, a third experienced examiner was consulted for adjudication. The two primary examiners showed concordant foveal localization in 28 of 31 eyes (90.3%). This procedure was implemented to minimize the potential influence of individual observer subjectivity on manual foveal centering.

Image analysis was performed using AngioTool64 Version 0.6a (02.18.14) (National Cancer Institute, NIH, Gaithersburg, MA, USA) [24]. An early version of this software was originally introduced by Zudaire et al. to quantify vessel branch points per unit area, termed the “branching index,” which represents the ratio of branching points to the analyzed area. The finalized tool, AngioTool, extends these capabilities and allows assessment of a wider spectrum of vascular parameters. The software is well suited for the analysis of vascular plexuses [30,31].

**Figure 1:**
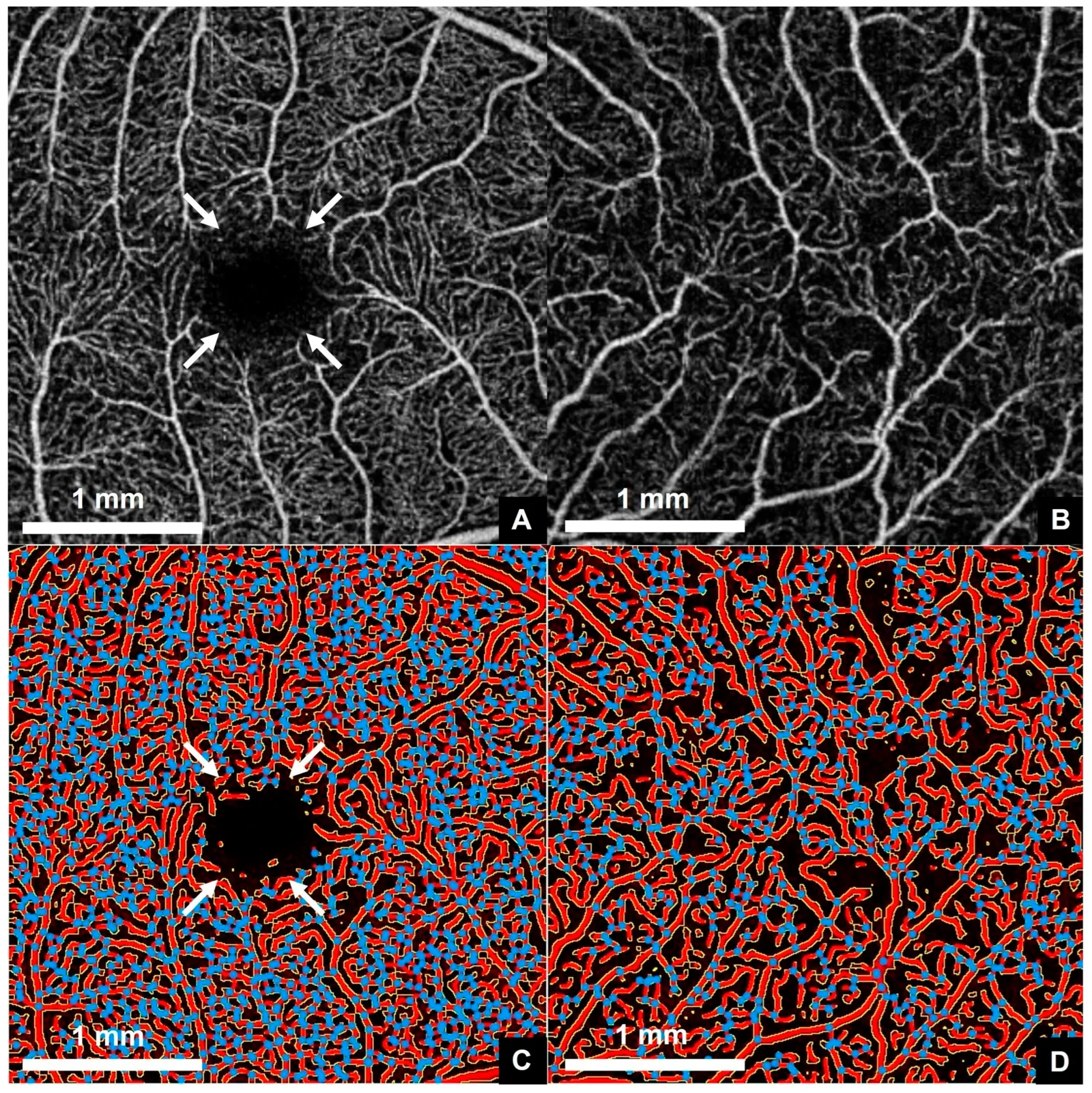
Exemplary AngioTool analysis of three-by-three mm^2^ whole en face OCT-A scans of a patient with FH and a healthy control. (**A**): Superficial capillary plexus (SCP), healthy; (**B**): SCP, FH (**C**,**D**): corresponding AngioTool analysis. White arrows indicate the foveal avascular zone (FAZ) in the healthy control eye. Scale bars represent 1 mm. In the AngioTool output, vessels are displayed with yellow outer vessel boundaries and a red vascular skeleton, while branching points are marked in blue. These color-coded overlays facilitate visualization and quantitative analysis of the retinal vascular network.

The software calculates multiple morphometric descriptors, including FD, number of junctions, total and average vessel length, vessel endpoints, and lacunarity. FD is defined as the proportion of bright pixels to the total scan area. Vessel length is represented by two measures: the total length, corresponding to the sum of all vessel segments within the network, and the average length, defined as the mean segment length between branching points. The number of endpoints was normalized to image size. Lacunarity, a measure of heterogeneity in vascular organization [32], was derived from variations in pixel mass density between vessel and background regions. Analysis was performed once the vessel mask overlay was aligned with the underlying image. Images of both the SCP and DCP were subsequently exported and processed with AngioTool (Figure 1).

In total, 31 eyes from 19 individuals diagnosed with FH and 31 eyes from 19 healthy controls were examined using OCT-A. For each patient with FH, a control subject of the same gender and comparable age (difference not exceeding three years) was recruited. Scans affected by artifacts were excluded from analysis. To guarantee image quality, only scans with a signal strength index (SSI) of 50.0 or higher were considered eligible. Each macular slab was acquired in three consecutive OCT-A scans. For statistical evaluation, the image with the best SSI was selected.

All FH cases included showed bilateral manifestation, confirmed by fundoscopy and multimodal imaging (OCT and OCT-A). In seven FH patients, only one eye met the predefined inclusion criteria. Consequently, the corresponding control eye was excluded prior to statistical analysis.

To assess the potential influence of FH severity on vascular parameters, subgroup analyses were performed between grades 1 and 2, which retain cone photoreceptor specialization (photoreceptor specialization present; PRS^+^), and grades 3 and 4, which lack this feature and are therefore classified as PRS^−^ (photoreceptor specialization absent) [2].

### Statistical Analysis

Dataset preparation was completed in Microsoft Excel (Microsoft, Redmond, WA, USA; 2010), and statistical analyses were performed using SPSS (IBM SPSS Statistics 28).

A priori sample size calculation, based on a two-sided independent-samples test with α = 0.05, a power of 80%, and assuming a large effect size (Cohen’s d = 0.8), indicated that a minimum of 25 eyes per group would be required. As the study included 31 eyes per group, the sample size was considered sufficient to detect large effect sizes with adequate statistical power.

Normality of distribution was assessed using the Shapiro–Wilk test. As the data were not normally distributed and groups were independent, comparisons were conducted using the nonparametric Mann–Whitney U test. To account for intra-individual clustering in cases where measurements from both eyes of the same patient were included, the test was modified according to the method of Rosner et al., which adjusts the variance to correct for within-subject correlations and has been specifically developed for ophthalmic datasets containing paired-eye observations [33]. This approach was chosen to allow inclusion of all eligible eyes while appropriately accounting for the lack of statistical independence between fellow eyes. Data are presented as medians with interquartile ranges (25th–75th percentiles). A significance level of *p* < 0.05 was applied.

Effect sizes were calculated using Cliff’s delta (δ) to quantify the magnitude and direction of between-group differences independent of sample size. Cliff’s delta ranges from −1 to +1, with negative values indicating lower values in the first group and positive values indicating higher values in the first group. For the primary analysis, δ was calculated for each AngioTool parameter comparing FH eyes with control eyes; for the exploratory subgroup analysis, δ was calculated comparing PRS+ and PRS− eyes. Effect sizes were interpreted according to established thresholds for Cliff’s delta. Given the absence of established clinically meaningful thresholds for the investigated OCT-A parameters in FH, effect sizes were considered measures of statistical magnitude rather than direct indicators of clinical relevance.

To account for multiple comparisons, the Benjamini–Hochberg procedure was applied to control the false discovery rate (FDR). For the primary analysis, the 12 comparisons of angiographic parameters between FH and control eyes across the SCP and DCP were considered as one family of tests. For the secondary, exploratory analysis, the 12 comparisons between PRS+ and PRS− eyes across both plexuses were considered as a separate family of tests. Both raw and FDR-adjusted *p*-values are reported. In light of the study’s exploratory design and small sample, the findings ought to be viewed as providing a basis for generating hypotheses rather than as evidence for definitive conclusions.

## 3. Results

Baseline characteristics of the study cohort are presented in Table 1.

The distributions of sex and laterality were balanced across groups. Disease severity was bilaterally symmetric in all but two individuals. In these exceptions, one eye was classified as grade 2, whereas the fellow eye was graded 3 or 4, respectively. VA was markedly better in the control group compared with the FH cohort (*p* < 0.01). In contrast, neither spherical equivalent (*p* = 0.40) nor SSI (*p* = 0.07) differed significantly between the groups.

The distribution of FH etiology across Leicester grades is presented in Table 2.

The FH cohort comprised 12 patients with idiopathic FH, six with ocular albinism, and one with oculocutaneous albinism. Idiopathic FH was represented across all Leicester grades, whereas ocular and oculocutaneous albinism occurred exclusively in grades 2–4.

### 3.1. Main Outcome: Angiographic Parameters

Quantitative assessment of the SCP revealed pronounced differences between FH and control eyes. Eyes affected by FH showed reduced FD, fewer vascular junctions, shorter total and average vessel lengths, and an increased number of endpoints. The corresponding effect sizes were moderate to large, with Cliff’s δ values ranging from −0.50 to +0.36. The largest effects were observed for the number of junctions (δ = −0.50) and total vessel length (δ = −0.46), whereas the effect for FD was moderate (δ = −0.33). The difference in lacunarity was smaller in absolute terms (δ = −0.36) and did not remain statistically significant after adjustment for multiple comparisons (Table 3).

In the DCP, eyes with FH exhibited significantly lower FD, reduced numbers of junctions, decreased total vessel length, and a higher number of endpoints. Effect sizes were generally small to moderate, ranging from δ = −0.36 to +0.37 for the significant comparisons. The largest effect size was observed for the number of endpoints (δ = +0.37), whereas the difference in average vessel length was negligible (δ = +0.14) and was not statistically significant (Table 4).

### 3.2. Secondary Outcome: Subgroup Comparison

In the exploratory subgroup analysis according to FH severity, PRS− eyes showed lower FD, fewer vascular junctions, shorter total vessel length, and higher lacunarity in the SCP compared with PRS+ eyes based on the unadjusted analyses. The corresponding effect sizes were moderate to large (Cliff’s δ = +0.47 for FD, +0.48 for junctions, +0.48 for total vessel length, and −0.50 for lacunarity). However, none of these differences remained statistically significant after Benjamini–Hochberg correction for multiple comparisons. No significant differences were observed for average vessel length or number of endpoints (Table 5).

Analysis of the DCP revealed no statistically significant differences in any of the evaluated parameters between the two groups (Table 6).

## 4. Discussion

While AngioTool has previously been applied to other macular vascular diseases, such as macular telangiectasia type 2 [25], this prospective study represents the first to combine OCT-A imaging with AngioTool software in FH. The main findings were significantly lower FD values, fewer junctions, shorter total vessel length and a higher number of endpoints in both the SCP and DCP of FH retinas after correction for multiple comparisons. Average vessel length was also significantly shorter in the SCP but not in the DCP. The initially observed difference in SCP lacunarity did not remain statistically significant after FDR correction. Exploratory subgroup analyses revealed lower FD, fewer junctions, shorter total vessel length, and higher lacunarity in the SCP in PRS^−^ compared with PRS^+^ eyes based on unadjusted analyses; however, none of these differences remained statistically significant after correction for multiple comparisons. No significant differences were observed between PRS^+^ and PRS^−^ eyes in the DCP.

FH is an ocular abnormality often associated with other systemic or ocular conditions such as albinism [2], followed by gene variants in PAX6 [3], SLC38A8 [4,5] and FRMD7 [6,7]. Prematurity is also a well-recognized risk factor [8]. A large cross-sectional study by Noval et al. involving 286 healthy participants, however, reported that up to 3% of children showed bilateral grades 1 or 2 FH [34], without the presence of an underlying condition. This finding suggests that a substantial number of cases remain undiagnosed, highlighting the importance of more accurate diagnostic approaches to improve our understanding of pathophysiological changes.

Compared with conventional FA, OCT-A offers several important advantages: it does not require dye injection and can visualize both the SCP and DCP. Consequently, it has become an invaluable diagnostic tool in retinal research [35]. In 2018, Sánchez-Vicente et al. published the first OCT-A case report of a patient with FH [12]. Given the low prevalence of FH and the relatively recent introduction of OCT-A, most published studies to date have remained limited to case reports [13,14], or very small case series with only two [15,16] or three patients [17,18,19], providing mostly qualitative rather than quantitative insights. The few quantitative OCT-A studies available report heterogeneous and sometimes conflicting results.

In our study, FD was significantly reduced in both SCP and DCP compared with healthy controls. These findings are consistent with the global OCT-A analysis reported by Le et al., who likewise observed significantly lower FD in whole en face scans of both vascular plexuses in eyes with FH [21]. However, they also reported increased FD in the central foveal region of the SCP, which at first glance appears to contrast with our findings. This discrepancy is likely explained by differences in the analyzed region of interest. Whereas Le et al. evaluated the central foveal area separately, our analysis focused on the vascular network across the entire en face image. In FH, the FAZ is absent or markedly reduced, allowing capillaries to persist within the foveal center. As a result, vessel density measurements obtained exclusively from the central fovea may be higher than those in healthy eyes. In contrast, whole-scan measurements predominantly reflect the abnormal vascular architecture and reduced vascular development of the surrounding parafoveal retina, leading to lower overall FD values.

These findings may indicate that vascular alterations in FH are spatially heterogeneous: local persistence of central capillaries may increase FD at the foveal center, whereas whole-scan measurements may reflect differences in vascular density and network organization in the surrounding parafoveal retina. Given the modest absolute differences observed for some parameters, however, the biological significance of these findings remains uncertain. This interpretation is further supported by developmental studies demonstrating that, despite incomplete foveal pit formation, compensatory retinal adaptations can occur in the central retina [36].

Total vessel length was markedly shorter in FH eyes across both the SCP and DCP. This parameter captures the cumulative length of all vessel segments within the network and is closely linked to FD. By contrast, average vessel length, defined as the mean length of individual vessel segments, was significantly shorter in the SCP but not in the DCP. This may indicate differential vulnerability of the two plexuses during foveal development. Our findings are consistent with those of Bazvand et al., who reported the absence of the FAZ in the SCP, while the DCP was affected only in some cases [19]. Similarly, Pakzad-Vaezi et al. found an intact SCP in the foveal area across all cases, but variable absence of the DCP, which was most pronounced in patients with poorer vision [23].

The number of vascular junctions was also significantly lower in FH eyes compared with controls. While this parameter has not yet been described specifically in the context of FH, it can be linked to the results of Storp et al., who were the first to extend investigations to macular subregions and deeper vascular layers including the choriocapillaris, as well as detailed FAZ morphology [22]. In their study of 19 FH eyes compared with controls, they reported significantly smaller FAZ size and perimeter and a higher acircularity index (ACI). Taken together with our findings, these results may be consistent with differences in vascular network organization associated with incomplete foveal development. The significantly higher number of endpoints observed in our study may further reflect differences in network architecture. However, these parameters describe structural features of the vascular network and should not be interpreted as evidence of clinically or biologically significant vascular impairment. In particular, the relatively modest absolute differences observed warrant cautious interpretation.

In the SCP, lacunarity was lower in FH eyes than in controls in the unadjusted analysis; however, this difference did not remain statistically significant after FDR correction. No difference was detected in the DCP. Lacunarity describes structural heterogeneity within a vascular network [32]. Although the initially observed lower values in the SCP could indicate a more homogeneous vascular arrangement, the lack of statistical significance after correction and the small absolute difference between groups warrant cautious interpretation.

To our knowledge, lacunarity has not previously been assessed in FH, and therefore, its biological and clinical relevance remains uncertain. Nevertheless, this parameter may provide complementary information regarding vascular network organization and merits further investigation in larger cohorts. In this context, our findings can be compared with studies evaluating fractal dimension, another measure of vascular network complexity based on methods such as box-counting or circular mass-radius analysis. Fractal dimension reflects the self-similarity of the capillary network and is widely used to quantify the complexity of biological structures. Le et al. reported reduced fractal dimension in FH eyes [21], suggesting alterations in vascular network complexity. However, whether differences in lacunarity are associated with disease severity, visual function, or clinical outcomes remains to be established. The absence of differences in the DCP could suggest relative preservation of deeper vascular architecture. Interestingly, Bhardwaj et al., in their OCT-A study of diabetic patients, observed the opposite pattern, with significantly lower fractal dimension in the DCP but not in the SCP [37]. These findings underscore the importance of analyzing both plexuses separately when assessing vascular changes.

When stratified by FH severity, PRS^−^ eyes showed greater alterations in vascular parameters within the SCP compared with PRS^+^ eyes in the unadjusted analyses. However, none of these differences remained statistically significant after correction for multiple comparisons. Thus, these findings should be regarded as exploratory and hypothesis-generating rather than evidence of a definitive association between FH severity and retinal vascular architecture.

Contrary to the findings in the present study, in a previous publication our group did not identify any significant association between OCTA metrics and FH grade [22]. However, it should be noted that our former analysis focused on different vascular parameters, namely FAZ morphology, rather than the quantitative metrics examined in the current study. Other case reports have described progressive adaptations in FAZ characteristics that correlated with disease severity [18]. Overall, the relationship between OCT-A findings and FH grade remains inconclusive. In the present study, no significant differences related to FH severity were observed in the DCP.

Although the observed differences reached statistical significance, their absolute magnitude was modest for some parameters. For example, SCP FD differed by approximately 2 percentage points between FH and control eyes (42.37% vs. 44.44%), corresponding to a moderate Cliff’s delta of −0.33. Similarly, SCP lacunarity differed only slightly in absolute terms (0.03 vs. 0.04), despite a moderate effect size (δ = −0.36); this difference did not remain significant after FDR correction. These findings illustrate that effect size and absolute numerical difference provide complementary information and should not be interpreted interchangeably. Importantly, no established thresholds currently define a clinically meaningful change in AngioTool-derived vascular parameters in FH. Therefore, the present effect sizes cannot be translated directly into clinical relevance or prognostic value. Rather, they indicate the magnitude of the observed between-group differences and may help inform future studies aimed at establishing normative ranges and clinically relevant thresholds.

Our findings suggest that OCT-A combined with AngioTool may provide valuable quantitative information on retinal microvascular alterations associated with FH. While AngioTool itself is an established image-analysis tool that has been applied to other macular vascular diseases, its use in FH allows quantitative characterization of vascular network features that have not been systematically investigated previously. The observed differences in vascular density and vessel architecture remained significant for most parameters after correction for multiple comparisons, whereas the observed difference in SCP lacunarity and the severity-related subgroup findings did not. Although these results indicate that OCT-A-derived metrics may contribute to a more detailed characterization of FH, the magnitude of some differences was modest and their clinical relevance remains uncertain. Therefore, the applicability of these metrics to disease grading, monitoring, prognostication, or clinical decision-making remains to be established. Future longitudinal studies with larger cohorts will be necessary to determine their diagnostic and clinical value and to define potential OCT-A-based reference ranges for FH.

### Limitations

This study has several limitations that should be considered when interpreting the results.

First, retinal imaging in individuals with FH is inherently challenging. Many affected patients present with nystagmus or photophobia, both of which can introduce motion artifacts and reduce the consistency of OCT-A acquisition. In addition, internal tracking algorithms depend on stable anatomical landmarks; the absence or distortion of structures such as the FAZ in hypoplastic foveas may hinder accurate alignment and further compromise image quality. Although ongoing advances in OCT-A technology—particularly in high-speed swept-source systems—are expected to improve motion robustness, reduce noise, and enhance structural detail, these limitations remain relevant in current clinical practice.

Despite these challenges, it is noteworthy that SSI did not differ significantly between the FH and control group in this study, which was achieved by careful image acquisition and image selection. Pradhan et al. recently emphasized the importance of SSI for inter-image comparability, particularly in the context of vascular density measurements [38]. Moreover, Lujan et al. demonstrated that higher SSI values are associated with better VA [39]. The absence of a significant SSI difference despite substantially higher VA in the control group therefore strongly supports the comparability of the two cohorts.

Second, the control group was selected retrospectively from a pre-existing institutional imaging database. Although controls were individually matched to FH patients for age and sex and were required to meet the same imaging quality criteria, retrospective selection may have introduced some degree of selection bias. Furthermore, matching for age and sex does not completely account for other potential confounding factors. Although relevant ocular and systemic exclusion criteria were applied, including the absence of ocular disease, previous ocular surgery, diabetes mellitus, and smoking, other factors such as differences in refractive characteristics, axial length, or other unmeasured systemic or ocular factors may still have differed between the groups. Spherical equivalent was assessed and is reported in the present study but was not included as an adjustment variable in the primary analysis. Therefore, residual confounding cannot be completely excluded and should be considered when interpreting the observed differences between FH patients and controls.

Third, the FH cohort was heterogeneous with regard to etiology, comprising patients with idiopathic FH as well as ocular and oculocutaneous albinism. These etiological subgroups may differ in their underlying developmental mechanisms and may therefore exhibit differences in retinal vascular architecture. As the number of eyes within the individual etiological subgroups was small, particularly for oculocutaneous albinism, meaningful statistical comparisons between etiologies were not feasible. The distribution of etiologies across Leicester grades was therefore assessed descriptively. Consequently, the present findings should be interpreted with caution with regard to their generalizability across different etiological forms of FH. Larger studies with sufficiently powered, etiology-specific cohorts are warranted to determine whether the observed vascular alterations are consistent across different causes of FH.

Fourth, multiple OCT-A parameters were assessed in both retinal vascular plexuses, and additional subgroup analyses according to FH severity were performed. To address the risk of inflated type I error rates, the Benjamini–Hochberg procedure was applied separately to the primary FH-versus-control comparisons and the secondary subgroup analyses to control the FDR. Although this approach reduces the likelihood of false-positive findings, it does not eliminate the possibility of type I errors, particularly given the limited sample size and exploratory nature of the study. Notably, several findings from the severity-based subgroup analysis that were statistically significant in the unadjusted analysis did not remain significant after correction. Accordingly, statistically significant associations should be regarded as preliminary findings that warrant validation in larger, independent cohorts.

Finally, the cross-sectional study design restricts us from commenting on temporal dynamics or progression of microvascular alterations in FH. Longitudinal studies are required to validate results displayed in this trial in order to assess severity-specific patterns of vascular involvement, and to better elucidate the underlying pathophysiological mechanisms in FH.

## 5. Conclusions

This prospective study provides a quantitative characterization of retinal microvascular alterations in FH using OCT-A and AngioTool analysis. Compared with healthy controls, eyes with FH showed lower FD, fewer vascular junctions, shorter total vessel length, and a higher number of endpoints in both the SCP and DCP after correction for multiple comparisons. Average vessel length was also reduced in the SCP, but not in the DCP. The initially observed difference in SCP lacunarity did not remain statistically significant after FDR correction, and severity-related differences between PRS^+^ and PRS^−^ eyes were not significant after adjustment.

These findings support the presence of alterations in retinal microvascular architecture in FH and provide further quantitative information on the vascular phenotype of the condition. However, the biological and clinical significance of these changes remains uncertain. OCT-A represents a useful complementary tool for characterizing FH, but its value for disease grading, monitoring, prognostication, or biomarker development requires validation in larger longitudinal cohorts.

## Figures and Tables

**Table 1:** General patient characteristics. Values are reported as absolute numbers and as median (25% and 75% quartiles).

	FH	Controls
*n* (Eyes)	31	31
*n* (patients)	19	19
age (years)	30 (16; 61)	30 (20; 61)
gender (M:F)	10:9	10:9
study eye (R:L)	16:15	16:15
median visual acuity (logMAR)	0.30 (0.20; 0.40)	0.00 (0.00; 0.10)
refraction (D):		
median spherical equivalent:	0.50 (−1.19; 2.81)	−0.25 (−1.44; 0.31)
median sphere	+1.50 (−0.50; 3.75)	0.00 (−1.00; 0.50)
median cylinder	−1.50 (−2.25; −0.88)	−0.50 (−0.50; −0.25)
median SSI	66 (62.5; 70.5)	68 (63; 78)

FH = Foveal Hypoplasia, *n* = number; M = male; F = female; R = right; L = left; logMAR = logarithm of the minimum angle of resolution; SSI = signal strength index.

**Table 2:** Distribution of foveal hypoplasia etiology across Leicester grading system stages. The number of eyes according to etiology and Leicester grade is shown. Percentages in the total column refer to the proportion of all included FH eyes.

Leicester Grade	Idiopathic FH	Ocular Albinism	Oculocutaneous Albinism	Total
1a	4	0	0	4 (13%)
1b	5	0	0	5 (16%)
2	4	2	1	7 (23%)
3	6	3	0	9 (29%)
4	3	2	1	6 (19%)
Total	22	7	2	31

FH = Foveal Hypoplasia.

**Table 3:** Results of the statistical comparison of AngioTool analysis of the superficial capillary plexus (SCP) between eyes with FH and control eyes. Values are presented as median (25% quartile; 75% quartile).

	FH	Controls	*p*	FDR-adj *p*	Cliffs δ
Flow Density (%)	42.37 (39.86; 44.29)	44.44 (42.06; 46.14)	**0.02**	**0.03**	−0.33
Junctions (*n*)	983 (870; 1102)	1166 (995; 1249)	**<0.01**	**<0.01**	−0.50
Vessel Length (µm)					
Total	24,614 (23,338; 25,989)	26,349 (24,753; 27,283)	**<0.01**	**<0.01**	−0.46
Average	278 (217; 435)	418 (343; 523)	**0.01**	**0.02**	−0.38
End Points (*n*)	565 (508; 688)	521 (482; 541)	**0.01**	**0.02**	+0.36
Lacunarity	0.03 (0.03; 0.04)	0.04 (0.03; 0.04)	**0.04**	0.05	−0.36

*p* values < 0.05 are highlighted in bold. *n* = number, µm = micrometer; FDR-adj = false discovery rate-adjusted; Cliff’s δ = Cliff’s delta.

**Table 4:** Results of the statistical comparison of AngioTool analysis of the deep capillary plexus (DCP) between eyes with FH and control eyes. Values are presented as median (25% quartile; 75% quartile).

	FH	Controls	*p*	FDR-adj *p*	Cliffs δ
Flow Density (%)	51.09 (49.03; 52.48)	52.13 (51.29; 53.55)	**0.01**	**0.02**	−0.36
Junctions (*n*)	1562 (1478; 1689)	1638 (1610; 1733)	**0.02**	**0.03**	−0.29
Vessel Length (µm)					
Total	29,814 (28,746; 30,766)	30,373 (30,021; 31,194)	**0.02**	**0.03**	−0.28
Average	936 (706; 1200)	844 (731; 1047)	0.59	0.59	0.14
End Points (*n*)	448 (406; 530)	412 (372; 448)	**<0.01**	**0.02**	0.37
Lacunarity	0.02 (0.02; 0.02)	0.02 (0.02; 0.02)	0.17	0.19	−0.30

*p* values < 0.05 are highlighted in bold. *n* = number, µm = micrometer; FDR-adj = false discovery rate-adjusted; Cliff’s δ = Cliff’s delta.

**Table 5:** Results of the statistical comparison of AngioTool analysis of the superficial capillary plexus (SCP) between FH eyes with and without cone photoreceptor specialization (PRS^+/−^). Values are presented as median (25% quartile; 75% quartile).

	PRS^+^	PRS^−^	*p*	FDR-adj *p*	Cliffs δ
Flow Density (%)	43.23 (42.32; 45.15)	39.98 (39.30; 42.78)	**0.02**	0.06	+0.47
Junctions (*n*)	1058 (965; 1149)	917 (748; 1006)	**0.02**	0.06	+0.48
Vessel Length (µm)					
Total	25,376 (24,335; 26,337)	24,157 (22,018; 25,143)	**0.02**	0.06	+0.48
Average	324 (247; 450)	232 (180; 384)	0.30	0.44	+0.37
End Points (*n*)	549 (508; 643)	661 (506; 754)	0.35	0.47	−0.15
Lacunarity	0.03 (0.03; 0.03)	0.03 (0.03; 0.04)	**0.02**	0.06	−0.50

*p* values < 0.05 are highlighted in bold. *n* = number, µm = micrometer; FDR-adj = false discovery rate-adjusted; Cliff’s δ = Cliff’s delta.

**Table 6:** Results of the statistical comparison of AngioTool analysis of the deep capillary plexus (DCP) between FH eyes with and without cone photoreceptor specialization (PRS^+/−^). Values are presented as median (25% quartile; 75% quartile).

	PRS^+^	PRS^−^	*p*	FDR-adj *p*	Cliffs δ
Flow Density (%)	50.41 (49.03; 52.93)	51.18 (48.79; 52.13)	0.57	0.62	+0.03
Junctions (*n*)	1618 (1495; 1727)	1548 (1459; 1671)	0.21	0.45	+0.28
Vessel Length (µm)					
Total	30,203 (28,860; 30,992)	29,367 (28,685; 30,625)	0.24	0.45	+0.28
Average	1025 (753; 1279)	931 (699; 1180)	0.47	0.57	+0.15
End Points (*n*)	468 (406; 518)	442 (394; 537)	0.76	0.76	+0.01
Lacunarity	0.02 (0.02; 0.02)	0.02 (0.02; 0.02)	0.29	0.45	−0.19

*n* = number, µm = micrometer; FDR-adj = false discovery rate-adjusted; Cliff’s δ = Cliff’s delta.

## Data Availability

The original contributions presented in this study are included in the article. Further inquiries can be directed to the corresponding author.
